# Anchylosis of the Lower Maxilla with the Temporal Bones

**Published:** 1842-05

**Authors:** 


*Anchylosis of the Lower Maxilla with the Temporal Bones.* Dr. Payan, surgeon to the Hôtel Dieu at Aix, in a collection of facts on different diseases of the head, which he has just published, reports this remarkable case. It seems that the patent, 77 years of age, was attacked by the cholera morbus in 1835, and carried to the hospital, where he died in two days. It was observed upon his admission that he could not move the lower jaw, and that the tongue was protruded through an aperture formed by the loss of the four upper incisor teeth. His children and relations stated, that during his lifetime he had frequently told them that he had been unable to move his jaw from between four and five years of age, which he attributed to an accident he met with at that time, viz: the fall of a table upon his head, probably causing fracture of the maxilla near to the condyles. Upon dissection, complete ossification was found to have taken place with both temporal bones, and the union was so perfect, that no line of demarcation could be seen between them. All the teeth existed, with the exception of one or two molar, and the four upper incisors which had been extracted to admit the introduction of food. They were in close apposition with each other. The inferior incisors were pushed more forwards, and the molars more outwards, than in the natural state. In each parotid duct was found a calculus. No trace of fracture could be discovered in any part of the bone. There was no anchylosis of any other joint. This curious specimen has been frequently exhibited by M. Dubreuil to the medical students at Montpelier.

Cases of anchylosis of this articulation have been recorded before, but they have generally been found only when other joints had undergone the same process. An officer died at

Metz in 1803, who had long been affected with general rheumatism, brought on by fatigue during the war, in a cold and moist country. Upon dissection, every articulation was found anchylosed, and his skeleton, which is now in the museum of the École de Médicine, forms in reality a single bone.—*Lond. and Edin. Monthly Jour. of Med. Sci.*, from *Gaz. Med. de Paris*, 28 Aug, 1841.

